# Enhancing Fetal Brain Imaging: ALPS-FMEG Technique Achieves Accurate Signal Extraction by Mitigating Movement Artifacts

**DOI:** 10.1007/s10439-026-03977-2

**Published:** 2026-02-05

**Authors:** Amer Zaylaa, Jürgen Dax, Katrin Sippel, Lorenzo Semeia, Joel Frohlich, Alban Gallard, Fabrice Wallois, Hari Eswaran, Andreas L. Birkenfeld, Hubert Preissl

**Affiliations:** 1 Institute for Diabetes Research and Metabolic Diseases (IDM) of the Helmholtz Center Munich, German Centre for Diabetes Research (DZD), 72076 Tübingen, Germany; 2 fMEG Center, University of Tübingen, 72076 Tübingen, Germany; 3 Internal Medicine IV, Department of Diabetology, Endocrinology and Nephrology, University Hospital of Tübingen, 72076 Tübingen, Germany; 4 MEG Center, University of Tübingen, 72076 Tübingen, Germany; 5 INSERM U1105, Université de Picardie, CURS Amiens, France; 6 Division of Maternal-Fetal Medicine, Department of Obstetrics and Gynecology, University of Arkansas for Medical Sciences, AR Little Rock, USA; 7 Institute for Advanced Consciousness Studies Santa Monica, CA, USA; 8 Department of Pharmacy and Biochemistry, Interfaculty Centre for Pharmacogenomics and Pharma Research, Institute of Pharmaceutical Sciences, Eberhard Karls University Tübingen, 72076 Tübingen, Germany

**Keywords:** Fetal neurodevelopment, Fetal magnetoencephalogram (fMEG), Independent component analysis (ICA), Empirical mode decomposition (EMD), Gross fetal movement detection, Fetal auditory event-related fields (fAEFs)

## Abstract

**Purpose:**

Fetal magnetoencephalography (fMEG) enables non-invasive monitoring of fetal brain function with high temporal resolution. However, how can we isolate low signal-to-noise ratio signals of the developing brain when disruptive artifacts arise from maternal and fetal movements? Addressing this challenge is critical for understanding brain development. We present Advanced Localization and Processing of fMEG Signals based on Maternal and Gross fetal body Movement Exclusion (ALPS-FMEG), a MATLAB-based framework that improves fetal brain signals by removing fetal and maternal movement artifacts.

**Methods:**

ALPS-FMEG integrates Independent Component Analysis for separation and reconstruction of fetal brain, fetal and maternal cardiac signal components in sensor space, Empirical Mode Decomposition for noise reduction, and a movement artifact detection-and-exclusion technique based on actogramCOG associated with heart rate patterns. This novel integration modifies the actogramCOG approach by pre-interpolating R waves for enhanced robustness and combines it with HRV-based logic gates, representing a first in fMEG processing to achieve artifact-free signals while preserving physiological latencies.

**Results:**

ALPS-FMEG was applied to 50 fMEG datasets from 28 to 39 weeks of gestation, enhancing signal quality. For group analysis, 45 datasets were retained after excluding recordings with auditory event-related field (fAEF) latencies < 70 ms. In these, it significantly improved signal-to-noise ratio and fAEF amplitudes (*p* < 0.0001), with preserved latencies. fAEF latency showed a significant negative correlation with gestational age (*p* < 0.001).

**Conclusion:**

ALPS-FMEG improves fetal brain signal extraction by addressing movement artifacts. This method supports robust fetal brain analysis and may be adaptable to future fMEG systems, including optically pumped magnetometers, enhancing prenatal neurophysiology and clinical research, though manual steps currently limit scalability and could be addressed via automation for broader practical use.

## Introduction

Understanding early brain development is vital for preventing maturational delays and developing effective intervention strategies for high-risk fetuses [1]. Fetal magnetoencephalography (fMEG) signals from the in-utero developing brain are overlaid with different interfering noise sources. The largest noise source after the maternal and fetal heart are movement-related signals from the mother and the fetus. Currently no effective method is established to eliminate movement-related artifacts in fMEG studies.

While fetal movements are essential for promoting muscle tone, skeletal growth, and neurological development [2], they pose a significant challenge in fMEG studies. These movements, though beneficial for fetal development, can introduce artifacts that obscure the brain signals being measured. This interference complicates the analysis of sensory processing and fetal brain evoked responses [3]. Despite comprehensive categorization of these movements in premature neonates [4] and fetuses [5], their impact on fMEG recordings remains a significant obstacle. Our study specifically targets gross fetal body movements, which are particularly disruptive to the quality of fMEG data.

Another challenge in working with fMEG data is uncertainty regarding the fetal brain position during the scan. Traditional techniques, such as orthogonal projection, have been employed to isolate fetal brain signals by excluding maternal and fetal magnetocardiography (mMCG and fMCG) signals [6, 7]. However, this approach often leads to the redistribution of residual fMEG signals among sensors. Hence, the reconstructed sources are not necessarily reprojected to the correct locations of the MEG channels, leading to spatial inaccuracies and difficulty in visual interpretation of the fMEG signal topography [7, 8]. Principal Component Analysis (PCA) has been applied to automate this process but has shown limited effectiveness [8], often resulting in attenuated fMEG signals and reduced detection accuracy [7]. In previous work from our group, only 19 of 45 available datasets could be robustly characterized by automated PCA-based analysis, with the majority failing criteria for reliable fetal brain signal detection [8]. This highlights the limitation of PCA in routine application for fMEG data, particularly for heterogenous fetal recordings. By contrast, although Independent Component Analysis (ICA) cannot fully eliminate source redistribution and requires manual intervention for grouping relevant sources components, it demonstrated superior capacity to isolate independent sources. It has been applied successfully in various fields, such as and not limited to EEG/ERP [9], EEG/fMRI [10], MEG [11], fMCG [12–15], fMEG evoked responses [16], and in neonate and fetal spontaneous MEG [17], demonstrating its efficiency even without full automation. In our context, this improved separation of different sources in fMEG data was necessary for meaningful comparison before and after movement exclusion, despite the labor-intensive nature of ICA. Therefore, and despite this limitation, we incorporated ICA as a core element of our proposed technique, since its proven ability to isolate fetal brain activity outweighed the drawback of increased manual effort of sources’ separation.

To ensure accurate grouping of fetal brain channels and then enhance the detection and processing of fetal brain signals, we have developed a novel technique called ALPS-FMEG which stands for Advanced Localization and Processing of fMEG Signals-based on Mother and Gross Fetal body movement Exclusion. What makes ALPS-FMEG particularly innovative is its adaptation of established tools into a unified MATLAB framework, including a modified actogramCOG (with pre-interpolation for uniform sampling) and HRV thresholds in a ± 5 s detection window, elements not previously combined in fMEG analysis. This is interesting as it not only improves signal quality but also uncovers maturational trends, such as reduced fAEF latencies with advancing GA, offering new insights into prenatal brain function. This approach begins by integrating Independent Component Analysis (ICA) to effectively group and extract signals from key sources, including the fetal brain, fetal heart, and maternal heart. Afterwards, we employ Empirical Mode Decomposition (EMD) to analyze the reconstructed brain signals, providing a more nuanced understanding of the data. Gross fetal movements are then detected using the actogram center-of-gravity (actogramCOG) technique introduced by Govindan [18]. We further refine this process by incorporating a combined analysis of the fetal actogramCOG with heart rate acceleration and deceleration parameters, which is adapted from Vairavan’s work [19], alongside the maternal actogramCOG. This integration is expected to enhance detection of subtle fetal movements missed by actogramCOG alone.

We hypothesize that systematically detecting and excluding periods of gross fetal and maternal movements during fMEG records, by means of ALPS-FMEG, will significantly improve the quality of brain signal recordings, particularly in the analysis of fetal auditory event-related fields (fAEFs). This advancement in fMEG methodology has important implications for enhancing our understanding of fetal brain function and improving the accuracy of neurodevelopmental assessments. By refining how we analyze fetal brain activity, our approach may also contribute to better-informed research and clinical practices related to fetal development and, potentially, early interventions for high-risk pregnancies.

In the following sections, we will detail the methodology behind ALPS-FMEG on sample fMEG dataset, present the results of our analyses, and discuss the broader implications of our findings for fMEG research and fetal neurodevelopmental assessment.

## Materials and Methods

### fMEG

MEG records magnetic signals corresponding to electrical currents in biological tissue [20], which are not distorted by the different layers of biological tissue [21], making it uniquely suited for studying the magnetic fields generated in the fetal brain in utero [22, 23]. Although magnetic fields pass through biological tissues largely undistorted, practical factors such as maternal body mass and the distance between fetal brain and sensors can influence signal amplitude and quality. In this context, fMEG utilizes superconducting quantum interference device (SQUID) sensors renowned for their exceptional precision toward capturing the biomagnetic signals from the developing fetus.

**Fig. 1:**
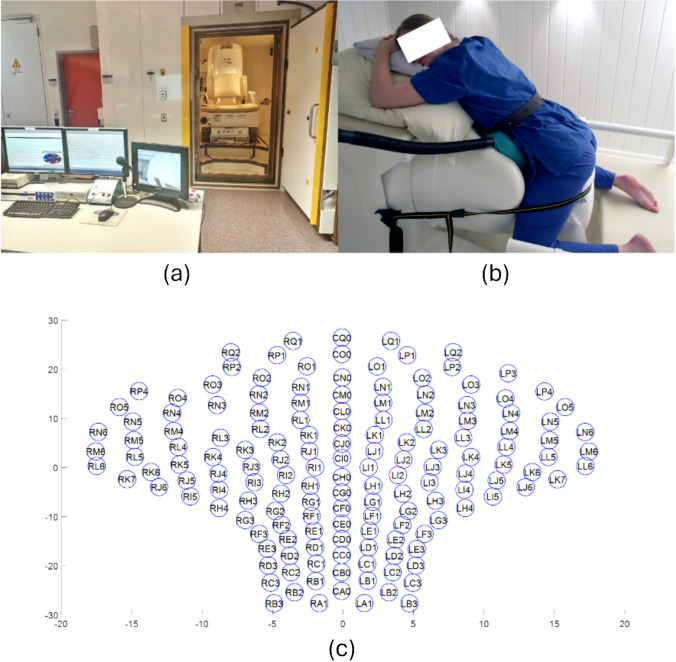
**a** External view of squid array for reproductive assessment (SARA) MEG system with its operating and monitoring console. **b** Common positioning of pregnant women on SARA device prior to a fetal MEG recording session. The green balloon on the left side of the chamber is used in fMEG with auditory stimulation recording session between the device surface and mother abdomen. **c** SQUID MEG sensors’ label distribution under the pregnant woman abdomen

The data acquisition is facilitated by the SARA (SQUID Array for Reproductive Assessment) system, located at the fMEG Center of the University of Tübingen (VSM Med Tech Ltd., Coquitlam, BC, Canada, see Fig. 1). To mitigate magnetic interference, the system operates within a magnetically shielded room (Vakuumschmelze, Hanau, Germany). The SARA system is equipped with an array of 156 primary magnetic sensors and 29 reference sensors conformed to the maternal abdomen to ensure optimal signal capture. Auditory stimulation is delivered through a balloon interface positioned between the maternal abdomen and the sensor array. Precise pre-recording and post-recording assessments of fetal positioning via ultrasound ensured the position of the fetal brain before and after the fMEG scan.

### Data

This comparative analysis before and after ALPS-FMEG includes 50 fetal magnetoencephalography (fMEG) datasets. Of these, 44 datasets from two cohorts were in part previously reported in an earlier study that employed a different pre-processing methodology [8]. The gestational age (GA) in our current dataset ranges from 28 to 39 weeks. The distribution of the week gestational age (WGA) and corresponding number of N subjects (WGA (N)) is as follows: 28 (3), 29 (3), 30 (2), 31 (3), 32 (8), 33 (5), 34 (4), 35 (5), 36 (4), 37 (6), 38 (4), and 39 (3), as detailed in Table 1 (Appendix 1).

Ethical approval for both studies, with corresponding numbers 339/2010OB1 and 476/2008MPG1, was obtained from the local Ethics Committee of the Medical Faculty of the University of Tübingen. Participants provided written consent for their initial involvement and for the subsequent reuse of their data in further research. Both studies employed an identical auditory paradigm using an oddball design, with 6 and 10 min recording durations, respectively, consisting of a standard tone at 500 Hz and a deviant tone at 750 Hz, with the standard tone occurring 80% of the total tones’ trials [24]. Each tone lasted 500 milliseconds, with an inter-trial interval of 1500–2000 milliseconds.

### Methods

#### ALPS-FMEG

Our ALPS-FMEG technique integrates several established robust signal processing methods, including ICA, EMD, fetal and maternal actogramCOG, and heart rate pattern analysis. We begin by defining the following terms for fMEG data labeling used throughout this study:

- ${fMEG}_{ori}$: Raw fMEG data acquired from SQUID MEG sensors, representing a mixture of biomagnetic signals from the maternal abdomen.
- ${fMEG}_{filtered}$: Bandpass-filtered version of ${fMEG}_{ori}$.
- ${fMEG}_{recon}$: Reconstructed fetal brain data derived by grouping ${fMEG}_{filtered}$ sources.

#### ALPS-FMEG Pre-processing Pipeline

**Fig. 2:**
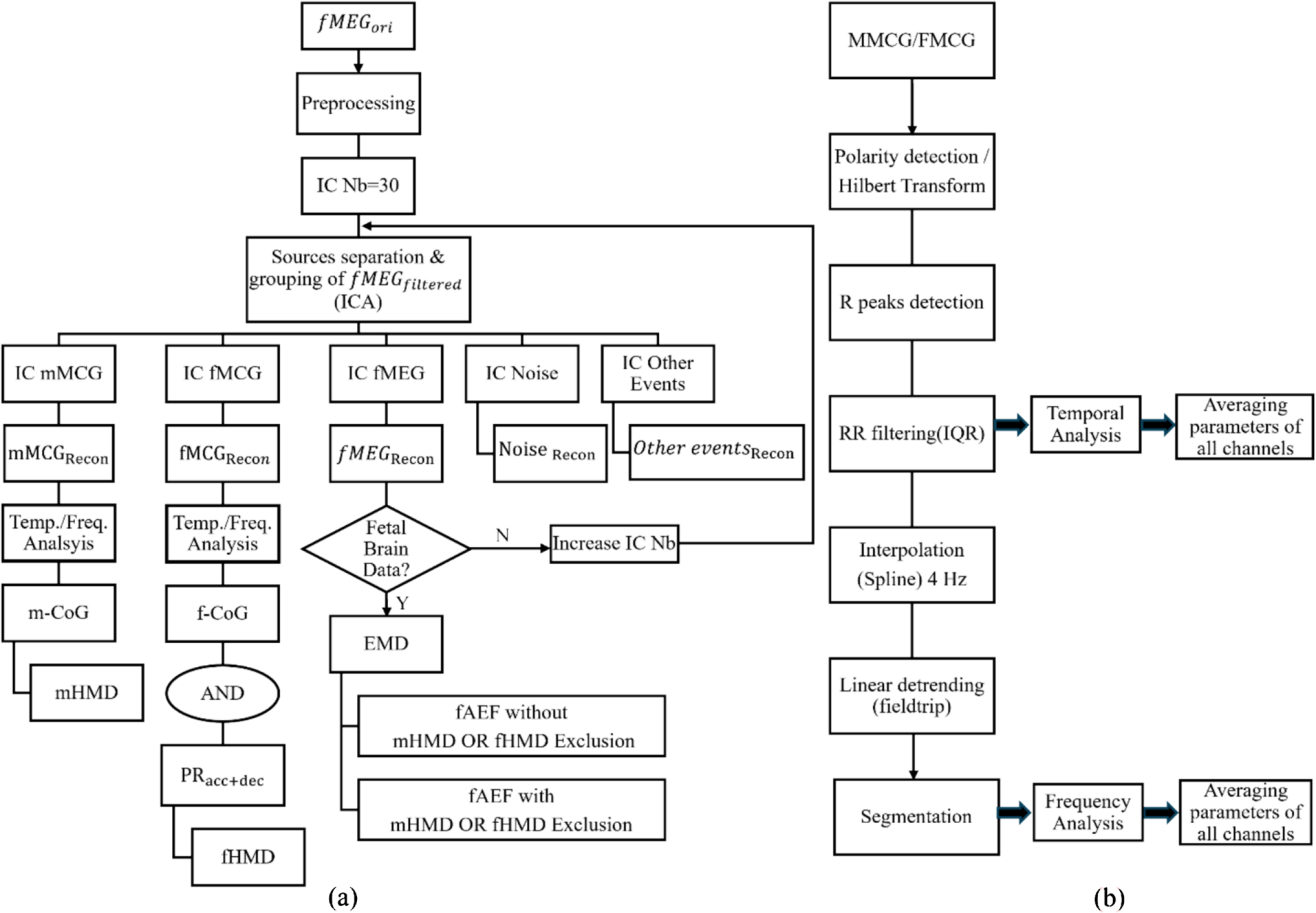
Pipeline flowchart of ALPS-FMEG (Advanced Localization and Processing of fMEG Signals-based on Mother and Gross Fetal body movement Exclusion) and detection of heart rate variability parameters (HRV). **a** Pipeline for processing the AEF with and without exclusion of fetal and mother movement after grouping the raw fMEG data using ICA into mMCG, fMCG, fMEG, noise and other events. **b** Pipeline of temporal and frequency heart rate variability (HRV) parameters’ extraction from mother and fetal MCGs [25]. ICA independent component analysis. IC independent components, mMCG maternal magnetocardiogram, fMCG fetal magnetocardiogram. fMEG fetal Magnetoencephalogram. Other events refer to the remaining biological signals such as uterine muscular activity signals. Recon: reconstruction, m-COG and f-COG: mother and fetal heart R-wave center of gravity over time respectively, based on [18]. ${PR}_{acc+dec}$: percentage of fetal heart rate acceleration and deceleration over time. EMD: empirical mode decomposition. AEF: auditory event-related field, mHMD mother heart movement detection, fHMD fetal heart movement detection

Figure 2a illustrates our data pre-processing and processing pipeline for computing Auditory Event-related Fields (AEFs) with and without exclusion of maternal and gross fetal movement artifacts from ${fMEG}_{Recon}$. Figure 2b depicts our method for extracting temporal and frequency parameters from mMCGs and fMCGs. We start our pre-processing as follows:

A. Bandpass Filtering:
  Data are initially subjected to a bandpass filter with a range of 0.8–100 Hz using a third-order Butterworth filter [25], combined with a 50 Hz notch filter to eliminate line noise. The filtering is extended to cover a broad frequency range to assess the robustness of our methodology against various signal components.
B. Visual Inspection:
  Post-filtering, data undergoes visual inspection using the FieldTrip rejection function to exclude channels with substantial variance from others [26]. Out of all the datasets, only one fMEG channel was marked as bad and excluded in Data 2 (Table 1 in Appendix 1). This step prepares the data for subsequent analysis using a blind source separation technique.

#### ALPS-FMEG with Independent Component Analysis (ICA)

ICA, a data-driven technique, seeks a linear representation of non-Gaussian data such that components are statistically independent [11, 27]. The ICA model is represented as follows:

1 $$\mathrm{S}={A}^{T}\mathrm{X}$$

Let us denote by *X* = ($${X}_{1},..{X}_{p})$$ a zero-mean p-dimensional random variable that can be observed, and by S = ($$1,\dots {S}_{p})$$ its p-dimensional transform. We wish to recover a constant mixing matrix A, which is a combination of $${a}_{ij}$$; *i* and *j* = 1,..*p*, so that the linear transformation of the observed variables are statistically as independent from each other as possible*.* In general, statistical independence, lack of sources correlation determines the entire cross moments (covariances) of a multivariate distribution [28]. By means of FastICA, we decompose the mixed signal,$${fMEG}_{filtered},$$ into its independent components. Here’s a simplified representation of the ICA model often used for $${fMEG}_{filtered}$$ analysis:

2 $${X}_{fMEG}={A}_{fMEG}*{S}_{fMEG}$$

where ${X}_{fMEG}$ represents the observed ${fMEG}_{filtered}$ (a matrix with multiple channels), ${A}_{fMEG}$ represents the mixing matrix, containing the unknown mixing coefficients that determine how the source signals are combined and ${S}_{fMEG}$ represents the matrix containing the independent source signals from mother heart, fetal brain, fetal heart and other biological sources.

The FastICA algorithm aims to estimate the inverse of the mixing matrix ${{A}_{fMEG}}^{-1}$ to recover the independent source signals (${S}_{fMEG}$). It was adapted to fMEG based on [11]:

A. Centering of ${X}_{fMEG}$ by substracting its mean vector; this implies that becomes zero mean as well ${S}_{fMEG}$ as ${X}_{fMEG}$.
B. Whitening the centered ${X}_{fMEG}$ by transforming the centered ${X}_{fMEG}$ linearly so that its components become uncorrelated and their variance equal unity. This can be done by computing the eigenvalue decomposition (EVD) of ${X}_{fMEG}$ covariance matrix.
C. Applying the FastICA learning rule, which finds a weight vector w such that the projection ${w}^{T}{X}_{fMEG}$ maximizes non-Gaussianity. Non-Gaussianity is here measured by the approximation of negentropy *J* (${w}^{T}{X}_{fMEG}$). The FastICA is based on a fixed-point iteration scheme for finding a maximum of the non-Gaussianity of ${w}^{T}{X}_{fMEG}$ [27, 29].
D. Computing the mixing matrix ${A}_{fMEG}$= ${w}^{T}$
E. Computing the different sources ${S}_{fMEG}$

In fact, we initially use 30 components, with the flexibility to expand them until the fetal brain data is isolated. This enables a manual categorization process of ${S}_{fMEG}$ into distinct 5 groups such as maternal heart, fetal heart, fetal brain, noise and other events including uterine smooth muscle activity, called magnetomyogram in [30] (Fig. 2a).

For manual categorization phase, each component is scrutinized for its 5 s temporal representation, frequency distribution, mixing matrix and its root mean square (RMS).

**Fig. 3:**
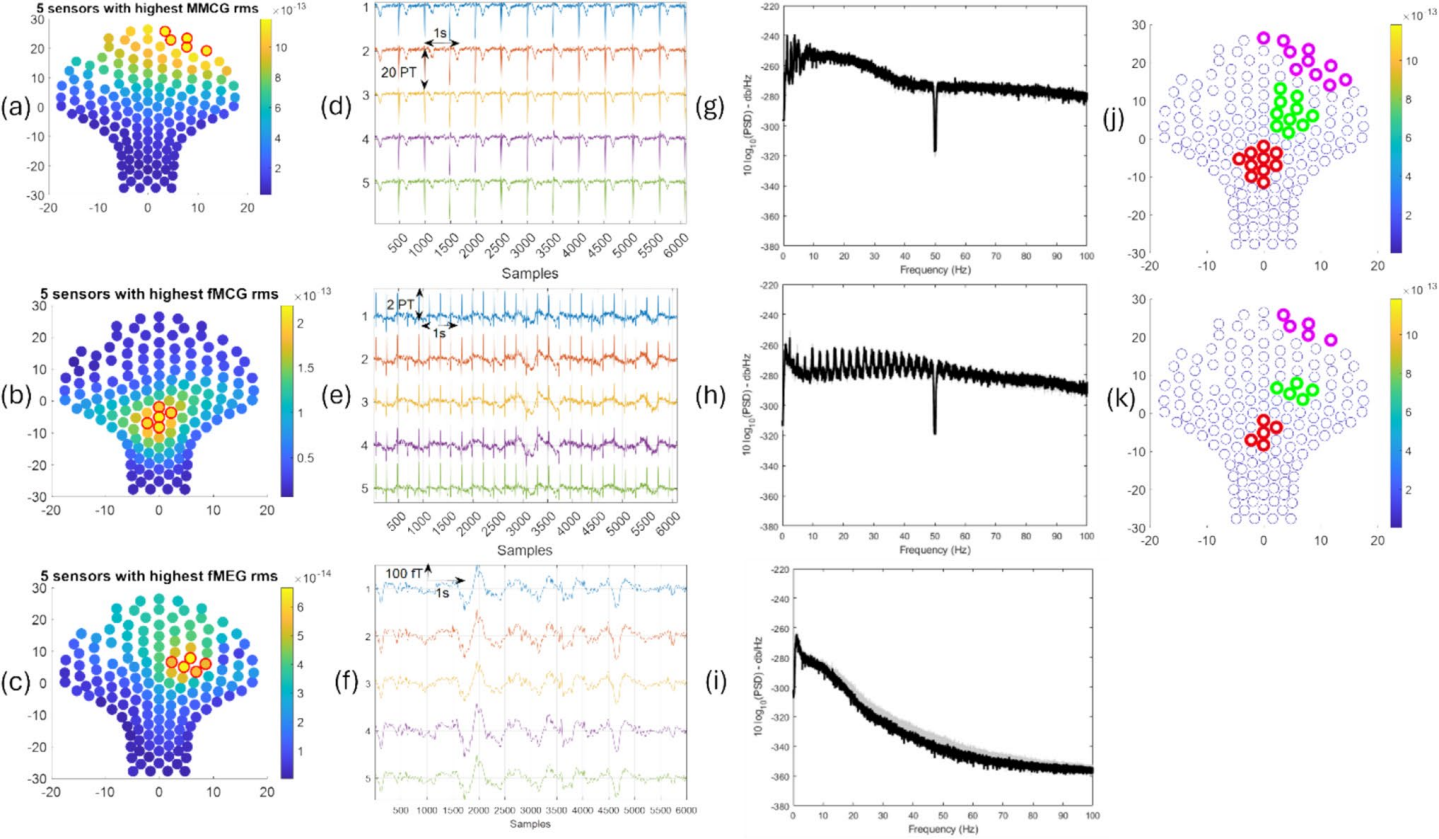
Localization of mother heart, fetal heart and brain of Data 47 with 35 WGA with their temporal and frequency distribution. **a**–**c** Selecting five sensors with high RMS (red circles) of reconstructed mother MCG, fetal MCG and fetal MEG, respectively. **d**–**f** 10 s temporal representation of reconstructed mother MCG, fetal MCG and fetal MEG, respectively, with 0.8–100 Hz IIR and 50 Hz notch filter. **g**–**i** Logarithmic frequency distribution of mother MCG, fetal MCG and fetal MEG, respectively. **j** Combination of high 10 RMS sensors allocating the mother heart (magenta circles), the fetal heart (red circles) and the fetal brain (green circles). **k** Combination of high 5 MMCG, fMCG and fMEG RMS sensors allocating the mother heart (magenta circles), the fetal heart (red circles) and the fetal brain (green circles)

Typically, the temporal representation reveals a more frequent R peaks for fMCG compared to mMCGs. The frequency distribution analysis examines the presence of harmonics, which are frequency components that are integer multiples of the fundamental frequency, for both fetal and mother MCGs, alongside the fundamental frequency itself. This analysis also checks for the 1/f distribution characteristic of fetal brain activity. Assessment of the ICA mixing matrix and its RMS space representation, like in Fig. 3a–c, facilitates the identification of regions with heightened activity in the 2D distribution, which is useful for distinguishing and grouping components. Based on this grouping, signals are reconstructed for each category using the linear ICA equation. At this stage, we compute for each reconstructed signal the RMS of all channels and then select the 5 and 10 channels (expected to cover the spatial grouped region size based on the WGA) with greatest amplitude as shown in Fig. 3a–c for mMCG, fMCG and fMEG, respectively. We show in Fig. 3d–f 10 s time-domain signal representations with a sampling frequency 610.3516 Hz as well their frequency distribution in Fig. 3g–i of mMCG, fMCG and fMEG, respectively. In Fig. 3i, the absence of a visually prominent 50 Hz notch is based on the lower spectral amplitude. A complete overview of the position of the maternal heart (magenta), fetal heart (red) and fetal brain (green) and thus their topology by means of grouping the selected 10 and 5 sensors with the highest RMS amplitude is illustrated in Fig. 3j and k, respectively. Indeed, this grouping is very important since additional criteria to the 1/f one, is added at this stage to confirm the fetal brain channels grouping by visually checking and looking for the fetal brain channels group near the fetal heart one. This allows us to analyze the fetal brain response in isolation and study its characteristics related to sensory processing and development.

#### ALPS-FMEG with Empirical Mode Decomposition (EMD)

EMD is a data-driven approach to decompose signal into intrinsic mode functions (IMFs) based on the variability of different frequency components. Huang et al. proposed this technique [31], which was combined later with canonical correlation analysis, a blind source separation, by Hassan et al. for denoising the uterine electromyogram (uEMG) [32]. Later on, it was applied in pre-processing phase of all acquired monopolar uEMG in the same laboratory group such as [33–35].

In the ALPS-FMEG pipeline, EMD is applied after ICA to further isolate fetal brain activity from residual noise. For each dataset, the relevant IMFs are manually selected based on their spectral and temporal characteristics. This manual selection ensures that components reflecting fetal brain responses, particularly auditory event-related fields (fAEFs), are retained while minimizing contamination from noise or overlapping physiological sources.

#### ALPS-FMEG with Mother and Gross Fetal Movement Detection and Exclusion

**Fig. 4:**
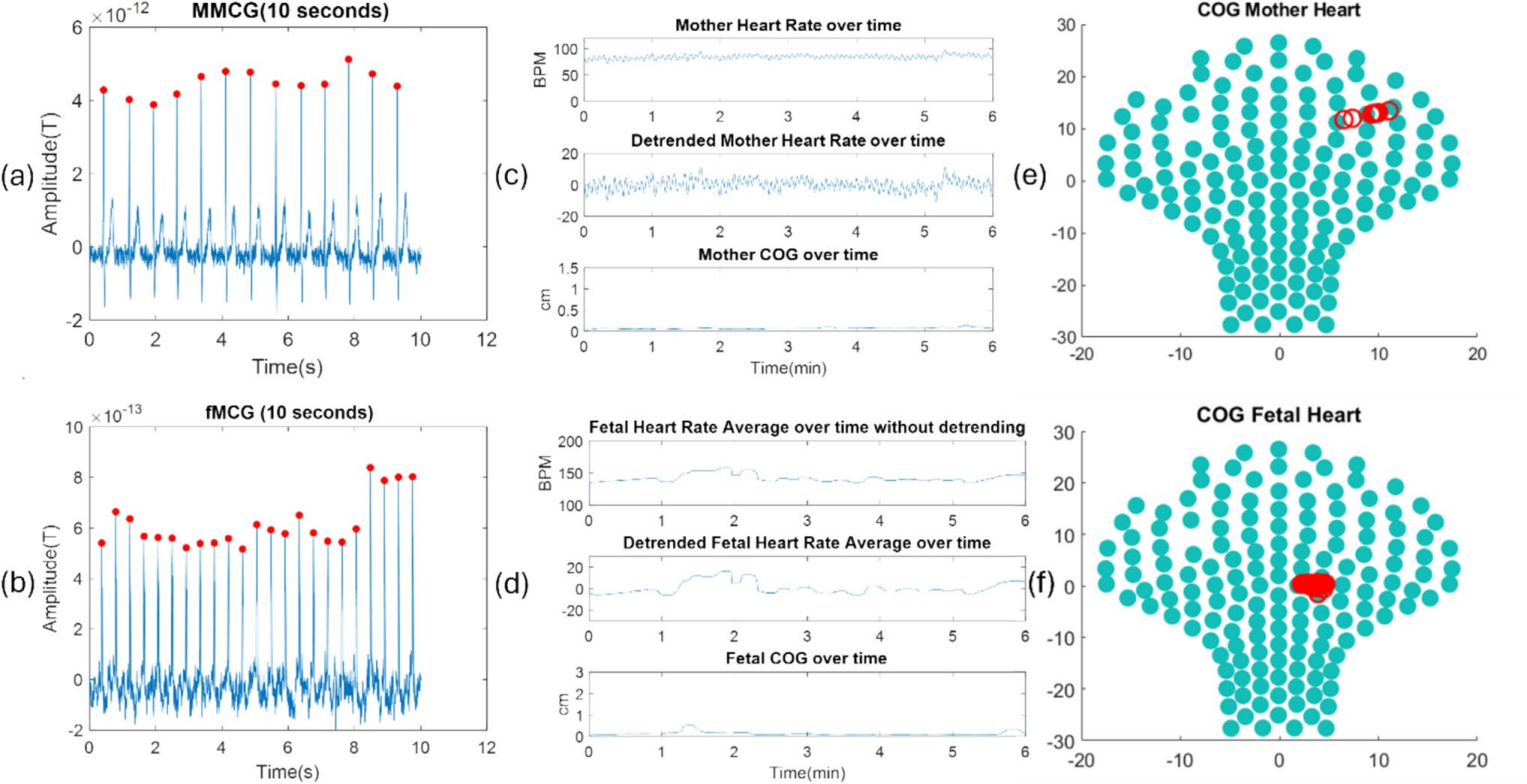
Mother and fetal heart rate and actogram of data 49 with 28 WGA. **a**, **b** A 10 s mother and fetal MCG respectively with detected R waves peaks depicted by red dots. **c**, **d** Mother and fetal heart rate, detrended heart rate and actogramCOG over 6 min session, respectively. **e**, **f** Mother and fetal COG localization on the SQUID MEG sensors’ space over the 6 min recording session, respectively
B. Maternal and Gross Fetal Movement Detection:
  We compute the actogramCOG as described by [18]. To avoid removal of physiologically relevant low-frequency variability we calculated for each filtered and interpolated R-wave prior to detrending the center of gravity (COG) based on Eq. 4, resulting in a three-dimensional vector. The actogram represents the RMS of the distance between the COG at each R-wave and the mean COG within the current 3 min window, following [5]. Figure 4c-3 and d-3 illustrates the COG for mother and fetus over a 6 min recording, with movement indicated by the position variations (red circles) in Fig. 4e and f.
  4 $${cog}^{j}=\frac{{\sum }_{i=1}^{N}\left({x}_{i}{y}_{i}{z}_{i}\right){|R}_{j}^{i}|}{{\sum }_{i=1}^{N}{|R}_{j}^{i}|}$$
  Following the original method that computes the actogramCOG using unevenly spaced R-wave occurrences before interpolation, we modified the procedure by interpolating the R-wave times beforehand to create a uniformly sampled time series. This modification facilitates downstream signal processing steps, such as smoothing and detrending, that require regularly spaced data points. These adaptations introduce novelty by enhancing downstream processing stability, making the pipeline more robust than the original uneven R-wave method. While this differs from the original sequence, the center-of-gravity calculation remains consistent, preserving physiological accuracy and improving the robustness of movement detection in our pipeline.
C. Heart Rate Acceleration and Deceleration:
  We measure the percentage of maternal and fetal heart rate accelerations and decelerations exceeding 10 beats per 1 min window by analyzing the detrended heart rate (Fig. 4c and d), this threshold is based on [5].
D. Gross Fetal Body Movement Detection:
  By implementing an AND logic gate which takes as input arguments: the fetal heart COG and the percentage of detrended fetal heart rate acceleration and deceleration, we detect gross body movement within + /− 5 s (Fig. 5d and e) by referring to [44] after applying 0.12 cm threshold for the first input gate based on [18] and for the second one, a threshold values of 1.85% if WGA < 36 and 1.533% if WGA ≥ 36 in a 1 min window adapted from 3 min threshold value in [19].

Our technique for detecting maternal and gross fetal movements leverages the actogramCOG, which is based on R-wave detection from ${fMCG}_{Recon}$ and ${mMCG}_{Recon}$. To achieve this, we extract relevant HRV parameters as outlined in [25], incorporating those essential to our technique. Below is a summary of our approach:

A. Pipeline for HRV parameters Extraction:
  As shown in Fig. 2b, we extract heart rate variability (HRV) parameters in both the time and frequency domain. First, we detect the R-wave using either polarity detection or Hilbert transform, where many previous fMEG studies were Hilbert transform based techniques [25, 36], followed by the Matlab ‘findpeaks’ function marking R peaks in mother and fetal MCGs (red points in Fig. 4a and b). If the sum of the peaks’ amplitudes in MCG surpasses those in the polarity-inverted (negative) MCG, the original MCG is deemed positive, based on the assumption that R-peak amplitudes are higher than other MCG wave components (Q and S waves). We then calculate the RR intervals (where RR intervals is the distance between two consecutive R waves) and filter them using our IQR (Interquartile Range) technique, retaining intervals that meet the criteria in Eq. 3.
  3 $$RR_{filtered} = \left( {{\mathrm{RRintervals}} \ge {\mathrm{Q1}}{-}k*{\mathrm{IQR}}} \right)\& \left( {{\mathrm{RRintervals}} \le {\mathrm{Q3}} + k*{\mathrm{IQR}}} \right)$$
  where Q1 and Q3 are the first and third quartile, respectively, IQR = Q3 − Q1 and *k* = 1.5 [37]. At this stage, we extract the HRV parameters in the time domain: mean heart rate, standard deviation of RR intervals (SDNN) associated with sympathetic and parasympathetic nervous system function, and root mean squared of successive differences of RR intervals (RMSSD) for parasympathetic function [38]. For HRV in the frequency domain, we interpolate the filtered RR intervals using a spline cubic function, remove linear detrending, segment the mother’s heart signal into 5 min windows based on [39] and the fetal signal into 2 min windows based on [40]. Then, we use the Pwelch Matlab (MATLAB R2022a Update 5 (version 9.12.0.2039608), The MathWorks, Natick, MA, USA) function [41, 42] to compute low frequency (LF), high frequency (HF) for primarily parasympathetic ANS, LF/HF ratio associated with sympathovagal balance, normalized LF for sympathetic and parasympathetic ANS, and normalized HF. The frequency bandwidths are mother (LF: 0.04–0.15 HZ) based on [39] and fetal (LF: 0.08–0.2 Hz, HF: 0.4–1.7 HZ) based on [38]. We then average the values across all channels. These average HRV metrics are reported for completeness and will be further analyzed in future work, specifically investigating HRV values from the MCG channel with higher RMS compared to average HRV values across MCG channels, to explore relationships with auditory stimulation, fetal maturation, movement, gestational age, and other covariates. In the current study, the primary focus is on the methodological workflow for movement detection and exclusion to optimize HRV metric reliability. Nonetheless, changes in HRV parameters changes in HRV parameters over WGA reflect the maturation of the fetal ANS [43].

**Fig. 5:**
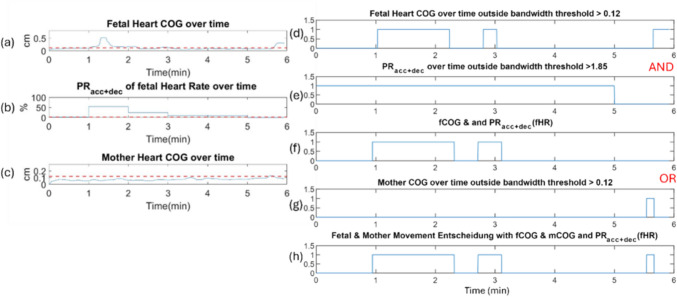
Detection of fetal and mother movement for Data 49 with 28 WGA. **a** Fetal heart COG over 6 min recording session. **b** Percentage of fetal heart rate acceleration and deceleration (${\mathrm{PR}}_{\mathrm{acc}+\mathrm{dec}}$) over 6 min recording session. **c** Mother Heart COG over 6 min recording session. **d** Logic decision of fetal heart COG higher than 0.12 cm. **e** logic decision of ${\mathrm{PR}}_{\mathrm{acc}+\mathrm{dec}}$ higher than 1.85% in 1 min block. **f** the logic decision of (**d**) AND **e** logic decision outputs, **g** Logic decision of mother heart COG higher than 0.12 cm. **h** The logic decision output of **f** OR **g** logic decision outputs. Dotted lines in **a**–**c** represent the threshold for each time series
E. Maternal Movement Detection:
  Similar to fetal detection, maternal movement is identified using a 0.12 cm threshold (Fig. 5g).
F. Exclusion of Movements:
  To exclude both maternal and gross fetal movements, we use the OR logic output of points E and F (Fig. 5h), thereby identifying and removing the movement time series.

#### ALPS-FMEG with AEF

**Fig. 6:**
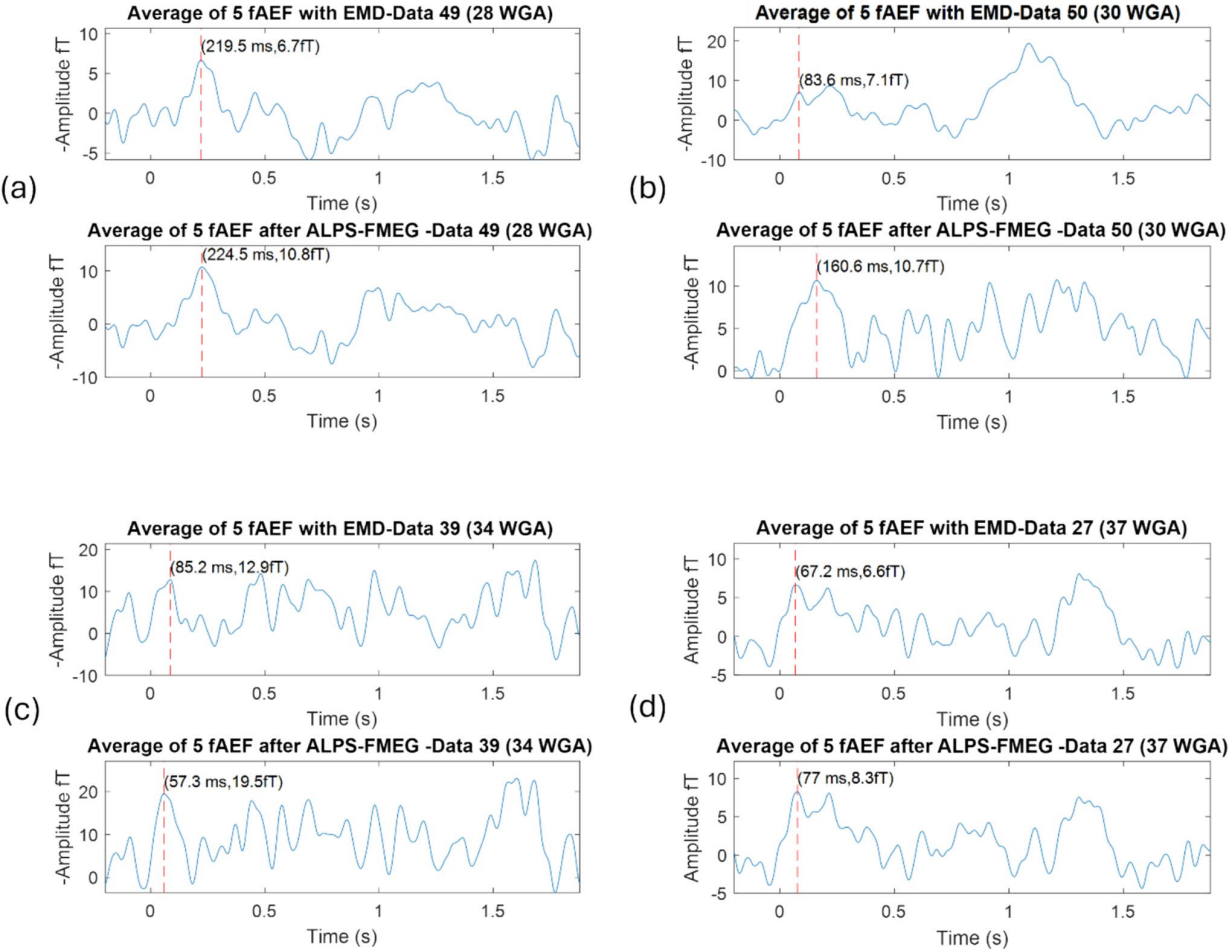
Fetal auditory events-related field (fAEF) analysis using ALPS-FMEG. **a**–**d** fAEF Average computed by averaging trials of 5 selected ${fMEG}_{Recon}$ channels of Data 49 with 28 WGA, Data 50 with 30 WGA, Data 39 with 34 WGA and Data 27 with 37 WGA before and after mother and gross fetal movement exclusion, respectively. Red dotted lines indicate key fAEF latencies and amplitudes (in time and amplitude) for Data 49, 50, and 27. The peak value observed at 57.3 ms in one dataset may be attributed to ear muscle activity rather than a true fAEF latency, as noted by Weitzman et al. who investigated the auditory event-related responses of premature infants [45]. Enhanced amplitude values are observed following the application of the ALPS-FMEG technique, demonstrating its effectiveness in reducing artifact noise

We first compute the AEFs for each of the 5 ${fMEG}_{Recon}$ channels by averaging all trials of standard tones. We define each trial as 2 s segment with 200 ms pre-stimulus baseline resulting in five AEFs for each dataset. Based on baseline evaluations, we chose to proceed with either the AEF or its inverse (-AEF) for latency and amplitude analysis. This approach ensures that the analysis is conducted using the signal with the correct polarity, accounting for potential polarity reversals due to fetal brain orientation. This adjustment enhances the reliability and accuracy of both latency and amplitude measurements. To ensure uniformity, we adjusted the polarity of any AEF channels that differed from the others, likely due to variations in the spatial positioning of certain grouped channels. Next, we identified the highest fAEF peak following stimulus onset within four predefined time windows using the Matlab function ‘findpeaks’: 0–199, 200–299, 300–399, and 400–499 ms, labeling these peaks as P1, P2, P3, and P4, respectively. Latency was determined by the time of the peak with the highest amplitude in these windows (Fig. 6). This study used a cross-sectional design: each dataset represents a single fMEG recording session from a unique pregnant participant. No individuals were measured multiple times, so statistical analyses were performed on independent samples. Data were checked for normality and analyzed by paired t-test for fAEF latency, latency peak amplitude and SNR (Signal-to-Noise Ratio) results before and after fetal and maternal movement exclusion. A statistical threshold of *p* < 0.05 was regarded as significant. The calculation was performed with Matlab R2022a.

#### ALPS-FMEG with SNR and Statistical Evaluation

Data were analyzed by paired t-test for amplitude and signal-to-noise ratio (SNR) results before and after fetal and maternal movement exclusion. A statistical threshold of *p* < 0.05 was regarded as significant. The calculation was performed with Matlab R2022a.

To calculate the SNR, we first corrected the noise baseline following the method outlined in Luck [46, 47]. Next, we computed the root mean square (RMS) of the signal after subtracting the average noise baseline within a 20 ms window centered around the peak latency; this windowing approach was based on the method described by Viola et al. [48]. The RMS signal value was then divided by the corrected noise baseline, calculated within the same time window before stimulus onset. Finally, we converted the resulting ratio to decibels using the formula:

5 $${\mathrm{SNR}} = 20{\mathrm{log}}_{10} \left( {\frac{{{\mathrm{Signal}}}}{{{\mathrm{Noise}}}}} \right)$$

where Signal and Noise refer to the RMS values computed for the latency-centered and pre-stimulus windows, respectively.

In addition, we performed two linear regression models with fAEF latencies and fetal gross body movement as dependent variables and WGA as independent one. This statistical approach is essential for understanding the fetal auditory developmental processes and evaluating if they follow a typical pattern or are affected by external factors as well as the second model.

#### Software and Tools

Data pre-processing, analysis, and statistical evaluations were conducted using MATLAB R2022a Update 5 (version 9.12.0.2039608, The MathWorks, Natick, MA, USA) and FieldTrip (version 20221101) [26].

## Results

### ALPS-FMEG Parameters Statistics

By applying ALPS-FMEG to the ${fMEG}_{Ori}$ dataset from 50 pregnant women (WGA ranging from 28 to 39), we were able to compute their fetal fAEF from five ${fMEG}_{Recon}$ channels each, with and without EMD and the exclusion of maternal and gross fetal movements. The averaged results are plotted in Fig. 6a–d for Data 49 (28 WGA), Data 50 (30 WGA), Data 39 (34 WGA), and Data 27 (37 WGA) following EMD and ALPS-FMEG processing.

We calculated the fAEF latency and amplitude based on the first peak with the highest amplitude after auditory stimulation onset, marked with a red dotted line in the figures. Latencies below 70 ms were excluded based on Weitzman et al. criteria who investigated the auditory event-related responses of premature neonates using EEG [45]. In this context, data from 5 subjects were excluded from our further fAER analysis.

A detailed illustration of all measured values is presented in Table 1 (Appendix 1), where “IC” denotes the number of FastICA components used to group for each dataset, ranging from 30 to 100. Additionally, the latencies ranged from 78.64 to 444.01 ms with an average standard deviation of 5.52 ms across channels before artifact exclusion. Post-exclusion, the latency ranged from 72.09 to 435.81 ms with an average standard deviation of 5.06 ms. The latency amplitudes ranged from − 0.49 to 16.3 fT with an average standard deviation of 0.656 fT across channels before exclusion and from 0.811 to 19.5 fT with a standard deviation of 0.831 fT after exclusion. The previous negative amplitude (− 0.49 fT) referred to the highest detected fAEF peaks in Data 44 indicating non detected fAEF latency before movement artifact exclusion.

SNR ranged from − 0.15 to 26.94 before artifact exclusion and from 4.43 to 49.41 after removing maternal and fetal movement artifacts. Gross fetal movement showed high variability, from 0% (no movement detected) to 88.78%, impacting the number of usable trials for fAEF computation. In this context, following artifact removal, the number of retained trials led to amplitude increases in various datasets. As illustrated in Fig. 6a–d, the peak amplitude increased as follows: for Data 49, it rose from 6.7 to 10.8 fT with 83 trials out of 128 standard tones; for Data 50, it increased from 7.1 to 10.7 fT with 81 trials out of 142 with a big shape’s variation in the fAEF affecting the latency detection value; for Data 39, it went from 12.9 to 19.5 fT with 84 trials out of 133; and for Data 27, it changed from 6.6 to 8.3 fT with 170 trials out of 200 standard tones before and after removing maternal and fetal movement artifacts.

Although HRV metrics were extracted for each recording and leveraged in the movement detection process to help identify and exclude periods of maternal and gross fetal movement, they were not statistically evaluated or reported as direct outcome measures in this study. Future analyses could investigate HRV parameters more extensively in relation to fetal brain activity and behavioral states, enabling deeper insight into physiological relationships beyond artifact exclusion.

### ALPS-FMEG with fAEF’s Latency, Latency Peak Amplitude and SNR

**Fig. 7:**
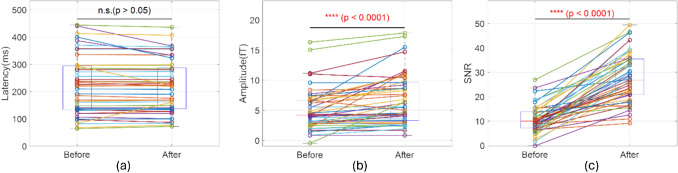
Box plots of fetal auditory evoked field (fAEF) latency and its amplitude, and SNR before and after maternal and gross fetal movement exclusion using ALPS-FMEG. Lines connect individual datasets (*n* = 45) before and after exclusion. P-values are indicated above the paired data for each parameter. **a** fAEF latency shows no significant difference before and after exclusion (*p*-value = 0.16). **b** A significant increase in amplitude is observed after exclusion (*p*-value = 2.07e-05). **c** SNR improves significantly after exclusion (*p*-value = 1.68e-15)

As for statistical analysis, a Kolmogorov-Smirnov (KS) test was performed on standardized data for each measure (fAEF latency, latency peak amplitude, and SNR), to evaluate normality. The results showed that the null hypothesis of normality was not rejected for any condition (all *h* = 0, *p* > 0.05), indicating that the data could be considered approximately Gaussian (Appendix 2). After excluding 5 dataset with fAEF peak less than 70 ms, the paired *t*-test results of 45 ${fMEG}_{Recon}$ dataset before and after mother and gross fetal movement exclusion of the fAEF latency paired *t*-test was not significant with *t*-statistic (44): 1.42, *p*-value = 0.16, confidence interval: [− 2.1e+00, 1.23e+01] as shown in Fig. 7a.

On the other hand, we show that the fAEF amplitude is significantly different before and after applying ALPS-FMEG in Fig. 7b with t-statistic (44): − 4.76, *p*-value: 2.07e-05, confidence interval: [− 2.56e-15, − 1.04e-15]. In addition, we illustrate the significance of fAEF’s SNR in Fig. 7c with t-statistic (44): − 12.02, *p*-value: 1.68e-15, confidence interval: [− 2.01e+01, − 1.43e+01].

While these paired t-tests show significant improvements in amplitude and SNR, the number of retained trials varies across datasets (Appendix 1, Table 1). Datasets with few remaining trials may exhibit less stable averages and potential overestimation of response amplitude due to sampling variability.

### Gross Fetal Movement and fAEF with WGA

Meanwhile detecting the gross fetal movement, we noticed a clear synchronization between heart rate evaluations and fetal actogram in Fig. 4d, with fetal heart rate accelerations coinciding with or preceding fetal movements nearly all the time.

**Fig. 8:**
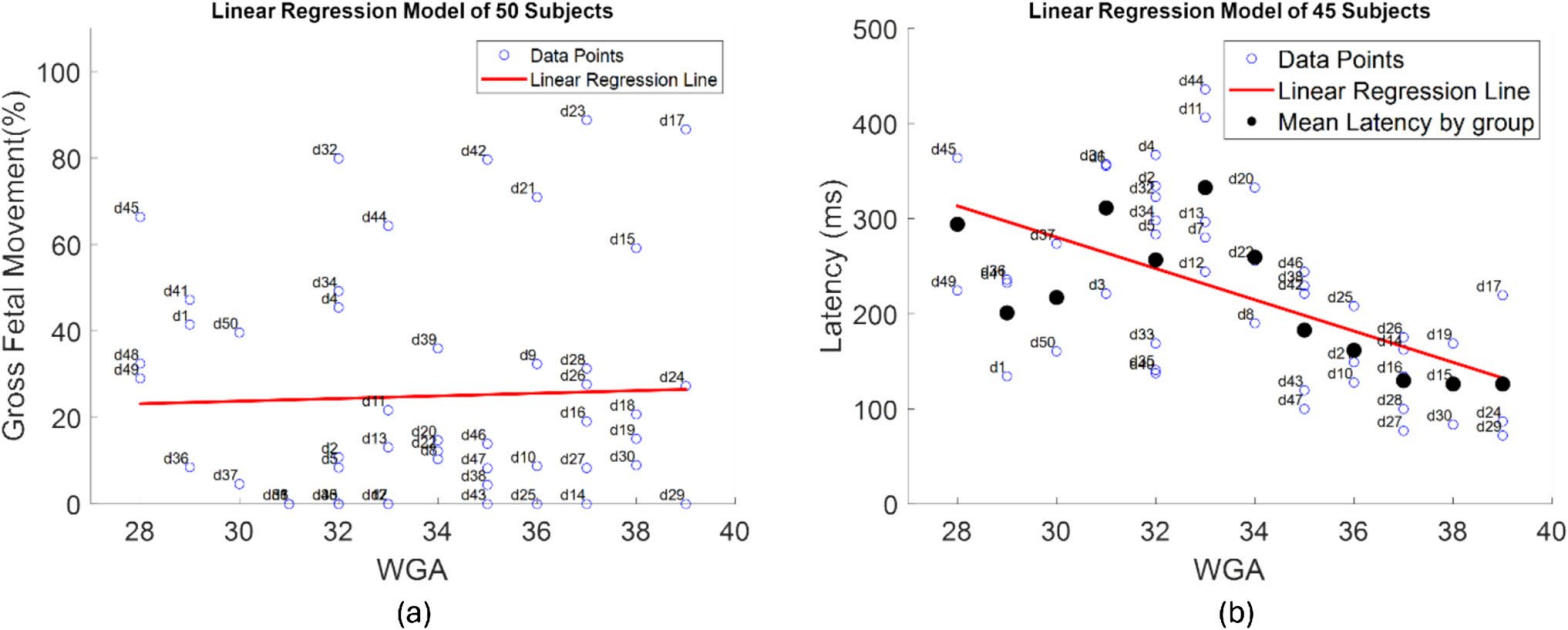
Linear regression model of gross fetal movement percentage and fAEF latency over week gestational age (WGA) **a** Linear regression model of gross fetal movement percentage with WGA for 50 fMEG dataset. F-statistic versus constant model: 0.064, *p*-value = 0.801, X estimate coefficient is 0.3. **b** Linear regression model of fAEF latency with WGA for 45 fMEG dataset, 5/50 subjects were excluded due to low fAEF peaks (< 70 ms). F-statistic versus constant model: 17, *p*-value = 0.000167, *X* estimate coefficient: − 16.44. The black points refer to the average fAEF latencies of data with 28, 29, 30, 31, 32, 33, 34, 35, 36, 37, 38 and 39 WGA, respectively

The gross fetal movement percentages range from 0 to 88.78% throughout the session duration (6-10 min), with an average of 25.41% and a standard deviation of 26.22%. Next, as we show in Figure 8a, the linear regression model of gross fetal movement percentage with WGA where observation number is 50, F-statistic vs. constant model: 0.064, *p*-value = 0.801, Root Mean Squared Error: 26.5 and X estimate coefficient is 0.3, which revealed no significant correlation. Furthermore, the linear regression model of fAEF latency with WGA is computed for the remained 45 participants in Fig. 8b with *p*-value: 0.000167, Root Mean Squared Error: 98, R-squared: 0.283, adjusted R-Squared: 0.267, F-statistic vs. constant model: 17, and with an X estimate coefficient of − 16.44 and illustrated a negative correlation. The black points, in both regressions, refer to the average fAEF latencies of data with 28, 29, 30, 31, 32, 33, 34, 35, 36, 37, 38 and 39 WGA, respectively.

## Discussion

Despite the importance of regular fetal movements as an indicator of well-being in utero [49], we know that movement artifacts have a negative effect on ${fMEG}_{Recon}$ processing. This study underscores the improved detection of fetal brain signals by excluding maternal and gross fetal movements.

To investigate our hypothesis: “Excluding maternal and gross fetal movement artifact from fetal brain time series will enhance the ${fMEG}_{Recon}$”, we developed a novel analysis approach called ALPS-FMEG, as fetal gross body movements have been shown to be particularly prominent in the second and third trimester [50].

Innovation of ALPS-FMEG technique lies in its hybrid, data-driven approach that goes beyond traditional methods like PCA or orthogonal projection by incorporating temporal exclusion based on physiological markers (e.g., HR accelerations coinciding with movements). This is particularly interesting for researchers, as it handles heterogeneous fetal data (e.g., varying movement percentages from 0 to 88.78%) and could extend to real-time applications, differentiating it from prior work focused solely on source separation.

In this context, it effectively groups and reconstructs the different sources of ${fMEG}_{filtered}$ using Fast ICA which has previously emerged as a strong competitor to factor analysis due to its reliance on the non-Gaussian nature of underlying sources [28]. Given the low SNR of the fetal brain data [51], which constitutes the major constraint to the application of fMEG using cortical fAEF due to a variety of factors including the magnetic signals generated by the fetal and maternal hearts that inevitably obscure a straightforward signal analysis [52], the grouping and localization of fetal brain signals poses the most critical challenge in implementing our technique. This necessitated pre-processing the data more thoroughly than what is typical for data recorded in other postnatal populations, e.g., EEG from premature neonates, whose electrode positions are well known, and behavioral states are relatively well classified. For each fetal dataset, we initially set the number of independent components (IC) to 30, performed the Fast ICA approach, and manually grouped the source components based on specific criteria, such as for fetal brain data: the grouped brain channels were near the grouped fetal heart channels in addition to the 1/f frequency distribution shape [8]. If these criteria were not met, we increased the number of IC components and repeated Fast ICA until we identified the fetal brain signals. In many datasets, the number of IC components reached 100, as shown in Table 1 (Appendix 1), which highlights a potential disadvantage due to the lack of automation and time-consuming pre-processing. However, we were able to identify fetal brain data confidently by the end of the process, ensuring the accurate localization of fetal brain and heart positions.

ALPS-FMEG also succeeded by integrating the EMD technique with ICA one to further denoise the ${fMEG}_{Recon}$. EMD was selected based on its successful application in prior uterine EMG studies within our group, while preliminary tests with wavelet methods informed this choice [35]. Its adaptive, data-driven decomposition is advantageous for the complex and variable nature of fetal MEG signals. Comparative validation of EMD and wavelet methods for fetal brain signal processing is an important direction for future work. Indeed, without applying EMD to our ${fMEG}_{Recon}$, accurately and automatically computing the fAEF latency peak becomes challenging. The presence of noise in the computed fAEF results in multiple surrounding peaks, obscuring the true latency peak.

Furthermore, we demonstrated the effectiveness of the ALPS-FMEG pipeline in detecting and excluding maternal and gross fetal body movements. This technique integrates the fetal heart rate parameters with fetal and maternal actogramCOG, adapting parameter thresholds to a 1-minute window based on the previous 3-minutes works by Vairavan et al. [19]. The final decision logic was derived through a sample-by-sample comparison of the combined parameters. We then computed and compared the fAEF with and without excluding trials that coincided with periods of maternal and gross fetal movements.

One surprising outcome, shown in and not limited to Data 50, was the change in the average fAEF shape from no detectable latency to a detectable one (Fig. 6b). This might address the difficulty in interpreting the absence of event related and mismatch responses in some fetal samples as noted by [53], potentially due to the difficulty of fetal brain signal detection [51].

An additional interesting outcome was revealed by Data 32 in Table 1 (Appendix 1): even after excluding 79.72% of the ${fMEG}_{Recon}$ time series, leaving only 19 out of 142 standard tones trials, the average fAEF showed a significant increase in amplitude from 6.2 to 15.4 fT.

From an electrophysiological perspective, during the implementation of the ALPS-FMEG pipeline, we observed synchronization between fetal actogram and heart rate evaluations, with fetal heart rate accelerations coinciding with or preceding fetal movements nearly all the time. This supports the hypothesis of coordinated control of fetal heart rate accelerations/decelerations and movement. In [44], Zhao et al. addressed the simultaneity of fetal heart rate acceleration onset and fetal trunk movement, concluding a decrease in coupling latency from 5 to 2 s over the last 10 weeks of pregnancy. Consequently, we adapted the ALPS-FMEG pipeline to detect gross fetal movement within a ± 5 s latency range when comparing fetal actogramCOG and heart rate changes, similar to the approach in [19], and this itself constitutes a novelty in this scientific research.

Regarding fetal neurological development, we computed the fAEF for the fetuses starting at 28 WGA by averaging only 500 Hz standard tones trials of the auditory stimulations with different frequencies (500 and 750 Hz usually employed to avoid fetal auditory system habituation in learning studies where the 500 Hz stimulations are more frequent in terms of number, around 80% of each recording session). The general approach of most fAEF studies is to search for an event-related component reflecting the latency of the primary response component and corresponding to the adult N100. Indeed, we referred our latency computation in this study to the work of Weitzman et al. on the auditory events-related response from premature infants by considering only the event-related components above 70ms as latency [45].

The average fAEF latencies demonstrated notable variability, with measurements ranging from 72.09(0) ms for Data 29 (39 WGA) at 0% gross fetal movement, to 435.81 (3.91) ms for Data 44 (33 WGA) at 62.58% gross fetal movement. The values in parentheses represent the standard deviations of five fAEF channels’ measurements. A plausible explanation for the notably low latency observed in Data 29 compared to Data 44 could be at first instance, the positioning or movement of the fetus, which might influence the mechanical coupling of auditory stimuli to the fetal auditory system [54]. However, this hypothesis requires further investigation, as it does not fully account for all variations observed across datasets. For example, in Data 14 (37 WGA), where there was also 0% gross fetal movement, the fAEF latency was detected at 162.2 ms, significantly longer than that of Data 29. This large variability in fAEF latencies could arise from several factors [54]. First, variations in fetal auditory system maturation across different gestational ages could contribute to differences in neural processing speeds. Additionally, the acoustic impedance of the amniotic fluid, variations in fetal positioning, and the degree of ossification of the fetal auditory structures might affect the latency of evoked responses. It is also possible that transient environmental factors during data collection, such as maternal physiological states, could influence latency measurements. To understand this neurological behavior, we performed a linear regression analysis of fAEF latencies across the remaining 45 dataset in relation to WGA. This regression analysis revealed a significant negative correlation with WGA indicating that as gestational age increases, fAEF latencies tend to decrease. This trend aligns with Schleussner et al. work findings [55] who highlighted the progressive maturation of the fetal auditory system and was later corroborated by Holst et al. [24]. While gestational age and fetal movement significantly influence fAEF latencies, other biological and methodological factors must also be considered to account for the observed variability. Further research focusing on the interplay between fetal neurophysiological maturation, acoustic properties of the fetal environment, and methodological factors during measurement is warranted.

Furthermore, a paired *t*-test comparing fAEF latencies (in milliseconds) before and after excluding movement artifacts yielded no significant difference (*p*-value: 0.16), suggesting that the removal of artifacts did not impact the latency time results.

Despite the non-clear correlation between the percentage of gross fetal movement and WGA, gross fetal movement percentages exhibited considerable variability throughout the session duration ranging from 0 to 88.78%. This variability provides preliminary evidence of fetal health and constituted the foundation for the classification of fetal behavioral states [2, 5, 19, 43]. Although behavioral state was not systematically evaluated in this study, future work should consider behavioral state classification as a factor influencing auditory evoked response latency, given its established impact on neurophysiological responsiveness in the fetus.

To prove the efficiency of ALPS-FMEG, we compared the fAEF amplitude and SNR before and after excluding maternal and gross fetal body movements. The paired *t*-test statistics were significant, allowing us to differentiate between the fAEF amplitude and SNR before and after movement removal (*p*-value < 0.0001 and *p*-value < 0.0001, respectively).

To conclude, we acknowledge that ALPS-FMEG currently requires manual input and expert intervention. While ALPS-FMEG advances fMEG analysis, limitations include manual components in ICA and EMD, which are time-intensive and require expertise, potentially hindering routine clinical adoption. The cross-sectional design and modest cohort size (*n* = 45 after exclusions) limit generalizability, and variability in the number of retained trials may introduce amplitude estimation biases. In clinical practice, these limitations could be addressed by automating source grouping via machine learning algorithms (e.g., clustering based on spectral or spatial features) and by validating the pipeline on larger, longitudinal datasets. Further integration with user-friendly interfaces or Optically Pumped Magnetometer (OPM) systems could enable real-time monitoring in prenatal clinics, facilitating early detection of neurodevelopmental alterations in high-risk pregnancies, such as those complicated by maternal diabetes. Ongoing efforts in our center focus on investigating fetal and neonatal behavioral states and testing the pipeline on larger datasets which will inform future steps aimed at increasing automation and enhancing usability for clinical applications. Our study contributed, via ALPS-FMEG, novel insights and solutions to mitigate the impact of both maternal and gross fetal body movements on fMEG data analysis. This consideration is crucial not only for examining fetal auditory fields, but also for analyzing broader event-related responses and spontaneous fetal brain activity. This framework could be adaptable for future integration with next-generation neuroimaging technologies, including portable systems based on OPMs, as the field moves toward more accessible fetal neuroimaging solutions. Clinically, ALPS-FMEG supports robust fetal brain assessments; however, to overcome current limitations such as manual processing requirements, future iterations could incorporate AI-driven automation and cloud-based tools for seamless integration into obstetric workflows, ultimately supporting personalized prenatal care.

## Figures and Tables

**Fig. 9:**
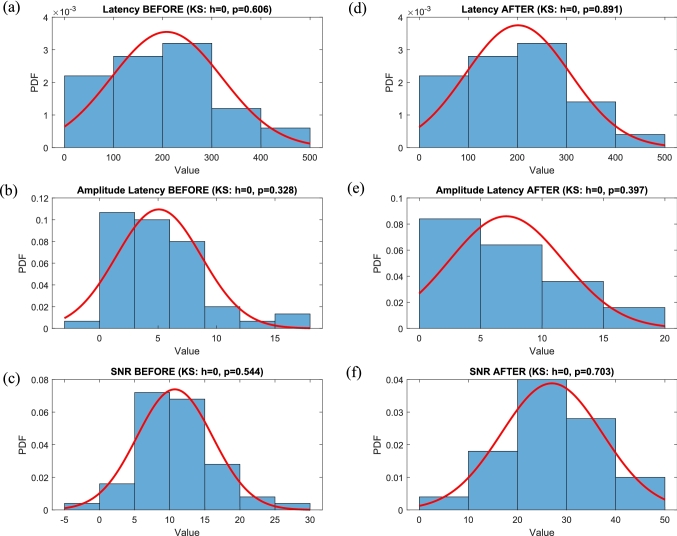
Histograms of fetal auditory event-related field (fAEF) latency, latency peak amplitude, and SNR before EMD and after ALPS-FMEG enhancement are shown in **a**–**f** respectively with overlaid probability density curves (kernel smoothing) to illustrate the approximate distribution, and Kolmogorov–Smirnov (KS) tests were performed on standardized data before running the *t*-test with the null hypothesis of normality not rejected for all conditions (*h* = 0, *p* > 0.05), with the corresponding h and p values indicated in each subplot

## Data Availability

Data are available in our database. We are open to share them for any collaborative research purposes. Requests will be considered to support transparent and impactful scientific reuse.

## Appendix 1

See Table 1.

**Table 1:** Fetal auditory event-related field latency and amplitude mean and standard deviation of 50 pregnant women ${fMEG}_{Ori}$ dataset before and after mother and gross fetal movement exclusion

D	GA	IC	fLME	fLSE	fLMA	fLSA	fALME	FALSE	fALMA	fALSA	SBE	SAA	GFMP	RT/TO
1	29	30	131.1	0.0	134.3	0.0	4.0	0.3	4.5	0.4	15.2	21.5	41.5	140/248
2	32	30	335.9	0.0	334.2	0.0	7.4	0.7	7.6	0.6	15.5	16.3	10.7	200/249
3	31	50	221.2	0.0	221.2	0.0	2.9	0.2	2.9	0.2	10.0	23.1	0.0	299/299
4	32	30	440.7	1.9	367.0	2.7	5.4	0.5	11.0	0.9	10.5	28.9	45.5	134/248
5	32	30	285.1	0.0	283.4	0.0	15.0	3.0	17.3	3.4	26.9	46.5	8.4	223/247
6	31	40	355.5	29.1	355.5	29.1	5.5	1.3	5.5	1.3	15.4	39.2	0.0	299/299
7	33	50	280.2	12.2	280.2	12.2	1.7	0.3	1.7	0.3	9.9	24.7	0.0	253/253
8	34	60	191.7	0.0	190.1	0.0	3.3	0.2	3.3	0.2	11.4	35.4	10.4	220/250
9	36	30	32.8	0.0	32.8	0.0	8.3	1.0	12.1	1.5	12.3	22.9	32.0	156/249
10	36	50	119.6	58.4	127.8	31.9	6.2	1.9	7.4	3.6	8.8	10.9	8.8	214/252
11	33	50	412.9	0.0	406.3	0.0	6.6	0.5	8.6	0.6	15.5	36.2	21.7	194/254
12	33	30	244.1	22.8	244.1	22.8	3.1	0.2	3.1	0.2	9.0	20.9	0.0	238/238
13	33	60	296.6	0.0	296.6	0.0	1.9	0.1	2.6	0.1	10.3	33.9	13.1	210/248
14	37	50	160.6	0.0	162.2	0.0	3.2	0.1	4.3	0.2	5.5	30.0	0.0	245/250
15	38	60	134.3	0.0	126.2	0.0	4.2	0.6	11.5	1.4	7.5	33.2	59.2	72/254
16	37	70	132.7	2.6	134.3	3.9	1.4	0.4	2.6	0.4	7.1	28.4	19.1	203/253
17	39	30	234.3	2.1	219.5	1.6	2.1	0.1	11.3	0.8	6.5	9.1	86.6	27/253
18	38	30	78.6	0.0	6.6	0.0	1.0	0.3	0.9	0.4	11.4	4.4	20.8	197/253
19	38	60	163.8	0.0	168.8	0.0	4.6	0.0	6.8	0.1	12.3	49.4	15.1	205/249
20	34	60	386.7	0.0	332.6	0.0	7.7	0.3	9.2	0.4	23.6	35.8	14.8	211/254
21	36	90	147.5	0.0	149.1	0.0	2.6	0.4	4.6	1.0	4.8	26.2	71.0	65/251
22	34	90	255.6	0.0	255.6	0.0	3.8	0.3	3.7	0.3	8.0	43.2	12.2	192/248
23	37	100	54.1	2.7	39.3	1.4	2.8	0.2	10.7	1.4	10.7	21.0	88.8	20/253
24	39	100	96.7	7.1	86.8	2.3	2.8	0.3	4.1	0.5	7.1	16.4	27.3	173/247
25	36	30	209.7	28.6	208.1	26.6	4.5	3.2	5.5	2.9	22.3	27.1	0.0	225/252
26	37	60	185.1	0.0	175.3	0.0	8.3	0.6	8.6	0.7	6.4	21.8	27.7	172/249
27	37	60	67.2	0.0	77.0	0.0	6.6	0.4	8.2	0.5	13.3	18.7	8.3	170/200
28	37	60	106.5	0.0	99.9	0.0	3.8	0.1	6.2	0.1	9.5	20.7	31.3	167/249
29	39	60	63.9	0.0	72.1	0.0	2.5	0.1	2.3	0.1	12.0	15.5	0.0	227/252
30	38	60	81.9	0.0	83.6	0.0	0.8	0.1	1.9	0.1	1.6	26.4	9.0	224/249
31	31	60	357.2	2.2	357.2	2.2	4.3	0.5	4.3	0.5	10.7	26.8	0.0	142/142
32	32	30	399.8	0.0	322.8	0.0	6.2	0.1	15.4	0.3	8.2	30.4	79.9	19/142
33	32	60	168.8	0.0	168.8	0.0	2.8	0.2	2.8	0.3	10.9	22.3	0.0	139/139
34	32	30	296.6	0.0	298.2	0.0	4.8	0.5	9.9	1.0	3.7	37.5	49.2	66/131
35	32	60	140.9	22.6	140.9	22.6	4.1	1.8	4.1	1.8	6.1	12.5	0.0	130/130
36	29	60	237.6	1.2	235.9	1.4	16.3	1.1	17.8	1.1	18.7	35.0	8.5	121/131
37	30	30	272.0	0.0	273.6	0.0	1.9	0.1	2.4	0.1	8.2	39.2	4.6	135/143
38	35	60	226.1	0.0	229.4	0.0	11.0	0.7	10.4	0.7	10.3	23.4	4.4	113/121
39	34	30	85.2	4.7	57.3	12.0	12.9	0.7	19.5	0.9	6.0	20.7	36.0	84/133
40	32	30	137.6	0.0	137.6	0.0	9.6	0.2	9.6	0.2	17.8	46.3	0.0	145/145
41	29	30	237.6	0.0	232.7	0.0	6.6	0.7	5.0	1.0	8.9	18.3	47.2	67/135
42	35	30	293.3	38.2	221.2	35.8	2.4	0.3	6.3	0.4	2.5	22.4	79.6	22/130
43	35	30	119.6	0.0	119.6	0.0	0.8	0.1	0.8	0.1	− 0.2	14.5	0.0	143/143
44	33	30	444.0	0.0	435.8	3.9	− 0.5	0.1	6.3	1.9	5.8	32.8	62.6	45/132
45	28	30	370.3	0.0	363.7	0.0	3.0	0.1	2.5	0.1	18.7	32.8	66.4	43/138
46	35	30	244.1	5.8	244.1	7.2	11.1	3.0	14.7	2.5	8.7	43.2	13.9	113/135
47	35	30	98.3	4.8	99.9	3.8	7.4	1.6	8.7	1.8	13.7	17.7	8.3	115/131
48	28	30	62.3	0.0	54.1	0.0	0.9	0.1	2.2	0.2	12.7	21.8	32.5	97/147
49	28	30	219.5	0.0	224.5	0.0	6.7	1.1	10.8	1.6	10.2	25.7	29.1	83/128
50	30	30	83.6	4.2	160.6	5.6	7.1	0.5	10.6	0.1	14.2	38.5	39.7	81/142
Total average (latency > 70 ms)			224.9	5.5	217.2	5.1	5.2	0.7	7.1	0.8	10.6	27.8	24.9	

*D* fMEG data number, *GA* pregnant woman’s week gestational age, *IC* IC components’ number used in ALPS-FMEG, *fLME* fetal auditory events-related fields (fAEF) latency mean after applying empirical mode decomposition (EMD) (ms), *fLSE* fAEF latency standard deviation after applying EMD (ms), *fLMA* fAEF latency mean after applying ALPS-FMEG (ms), *fLSA* fAEF latency standard deviation after applying ALPS-FMEG (ms), *fALME* fAEF amplitude of latency mean after applying EMD (fT), *fALSE* fAEF amplitude of latency standard deviation after applying EMD (fT), *fALMA* fAEF amplitude of latency mean after applying ALPS-FMEG (fT), *fALSA* fAEF amplitude of latency standard deviation after applying ALPS-FMEG (fT), *SBE* signal-to-noise ratio (SNR) before applying EMD, *SAA* SNR after applying ALPS-FMEG, *GFMP* gross fetal movement (%), *RT/TO* remained number of standard triggers trials after applying ALPS-FMEG/total number of original standard triggers trials before applying ALPS-FMEG

## Appendix 2

See Fig. 9.
